# Psychometric evaluation of the Norwegian version of the Threadgold Communication Tool

**DOI:** 10.1016/j.ijnsa.2025.100405

**Published:** 2025-08-06

**Authors:** Anne-Martha Utne Øygarden, Ellen Karine Grov, Anne Marie Mork Rokstad, Orla Brady, Knut Engedal, Benedicte Sørensen Strøm

**Affiliations:** a Oslo Metropolitan University, St. Olavs plass, 0130 Oslo, Norway; b Aldring og helse, Postboks 2136, 3103 Tønsberg, Norway; c University of Galway, University Rd, Galway, Ireland; d Lovisenberg Diaconal University College, Lovisenberggata 15B, 0456 Oslo, Norway; e Norwegian National Centre of Ageing and Health, Vestfold Hospital Trust; f Faculty of Health Sciences and Social Care, Molde University College

**Keywords:** Dementia, Psychometrics, Factor analysis, Statistical, Communication tool, SONAS

## Abstract

**Objectives:**

The study aimed to investigate the psychometric properties of the Norwegian version of the Threadgold Communication Tool, a proxy-rated instrument assessing communication abilities in people with dementia.

**Design:**

The study employed a prospective design, with two measurement points within 10 days. The Threadgold Communication Tool was translated into Norwegian following the World Health Organization's protocol for translation and back-translation.

**Setting and participants:**

The study included 126 residents from ten different nursing homes and one assisted living facility in Norway. The participants consisted of 99 women (78.57 %) and 27 men (21.43 %), aged between 61 and 100 years, with a mean age of 85.67 (SD 7.59).

**Outcome measures:**

The outcome measures were the internal consistency, test-retest reliability, and construct validity of the Threadgold Communication Tool.

**Results:**

The Norwegian version of the Threadgold Communication Tool demonstrated satisfactory psychometric properties, with a high level of internal consistency (Cronbach’s α coefficient = 0.95) and robust test-retest reliability (*r* = 0.8, *p* < 0.001). Principal Component Analysis revealed a two-component structure, explaining 62.9 % of the variance. However, the item 'vocalization' scored lower than other items and was identified as difficult to interpret by the Sonas Licensed Practitioners.

**Conclusions:**

The Norwegian version of the Threadgold Communication Tool is a reliable and valid tool for assessing communication abilities in people with dementia. However, further research is needed to refine the instrument based on these findings, particularly regarding the interpretation of the 'vocalization' item.

**What is already known**

• Communication is a fundamental human need, and assessing it in people with dementia is essential for person-centered care.
• Most communication assessment tools focus on behavioral issues like agitation rather than communication abilities.

Alt-text: Unlabelled box

**What this paper adds**

• This study demonstrates that the Norwegian version of the Threadgold Communication Tool has high internal consistency (Cronbach's α = 0.95) and strong test-retest reliability (r = 0.8).
• The study identifies that the item “vocalization” is problematic and difficult to interpret, suggesting a need for clarification or revision.
• This is the first psychometric evaluation to include the ‘smell’ and ‘taste’ items in the Threadgold Communication Tool, supporting their inclusion but also indicating potential redundancy.

Alt-text: Unlabelled box

## 1 Introduction

Dementia poses a substantial health challenge globally. According to the World Health Organization (WHO), it's estimated that dementia affects around 55 million people worldwide, with nearly 10 million new cases emerging each year. This number is expected to triple by 2050 (World Health Organization, 2023), highlighting the urgent need for research and intervention to tackle this escalating health issue.

In Norway, approximately 101,000 people are living with dementia, a number that is expected to rise to 236,789 in 2050, and to 380,134 in 2100. Furthermore, cognitive and functional impairments indicative of dementia are present in 80 % of long-term care nursing home patients, making it the most common reason for admission to such facilities (Norwegian Ministry of Health and Care Services, 2022). As Norway's elderly population ranks among the highest globally and continues to grow each year, the number of people over 80 is set to rise significantly in the future (Selbæk, 2023).

The sense of belonging and the wish to maintain social contact is a basic human need and does not change with dementia (Edvardsson et al., 2008; Eriksen, Engedal and Grov, 2022). Communication challenges among individuals with dementia are often constituted as one of the initial symptoms of cognitive impairment. As the disease advances, the capacity for communication, particularly verbal communication, diminishes (Engedal K, Haugen PK, Brækhus, 2005; Lykkeslet, Gjengedal, Skrondal, Storjord, 2014). Individuals with dementia often resort to non-verbal communication and behavior to compensate for the decline in verbal communication, as a means to maintain their engagement in the communicative world (Hubbard, Cook, Tester, Downs, 2002). However, these expressions may manifest as agitation, aggression, irritability, and repetitive vocalization (Engedal og Haugen, 2018), as well as through body movement, facial expressions, touch, physical appearance, vocal communication (Argyle and Dean, 1965), eye contact (Clare, Whitaker, Quinn, Jelley, Hoare, Woods, et al., 2012), or bodily conduct (Kontos, 2005).

Several instruments have been developed to evaluate various facets of communication. However, most of these tools primarily concentrate on the individuals' limitations and expressions, such as agitation and aggression, rather than their verbal or non-verbal abilities. To our knowledge, only two instruments are dementia-specific communication tools: the Holden Communication Scale (Holden and Woods, 1995) and the Threadgold Communication Tool (Strøm, 2018).

The Threadgold Communication Tool is a proxy-based instrument, primarily designed to assess communication abilities in persons with dementia after attending a Sonas Programme (described below). When the instrument was tested for reliability or validity in 2016, the tool had already been used extensively for about 25 years in nursing homes in Ireland. Initially, psychometrics was tested in 2016 in an Irish sample (*n* = 51) of people with dementia (Strøm, Engedal, and Grov, 2016). However, a Norwegian version of Threadgold Communication Tool was needed in relation to the implementation of the Sonas programme in Norway. Thus, the aim of this study was to investigate the psychometric properties of the translated version of the Threadgold Communication Tool in a Norwegian sample using the Norwegian version of the Threadgold Communication Tool.

### 1.1 Threadgold communication tool

The Threadgold Communication Tool is a proxy-rated instrument assessing communication abilities in people with dementia. It was developed in Ireland by Sr. Mary Threadgold, first in relation to the Sonas program. The instrument consists of 14 items, each graded from 0 to 4, from no evidence to frequent evidence of communication (Sonas aPc, 2011). The values measure observed communication abilities during Sonas group gatherings (described below). The higher the score, the greater the communication abilities. The highest cumulative point score is 56. Total scores below 14 indicate severe communication challenges, 15–25 indicate moderate communication challenges, 26–44 indicate mild communication challenges, and 45–56 indicate no communication challenges. In the initial psychometric evaluation of the English version of the Threadgold Communication Tool conducted by Strøm et al. (2016), the Cronbach’s α coefficient for the total score was determined to be robust, i.e. 0.95. The factor analysis yielded a two-component structure, explaining 73 % of the variance.

Numerous tools have been created to evaluate various aspects of communication. Most of these instruments predominantly focus on individuals' limitations and behaviors such as agitation and aggression, rather than their capacity to communicate, whether verbally or non-verbally. In a review by Robert et al. (2010), only one scale was identified for measuring communication abilities in people with Alzheimer's disease: the Communication Problem Scale. True to its name, this scale concentrates on communication-related limitations. Other scales, such as the Cohen-Mansfield Agitation Inventory (1989), primarily assess aspects related to agitation. Egan et al. (2010) highlighted in their review that only a few studies have addressed the communication interactions of people with dementia, recommending that future research should utilize tools reflecting a person's communication abilities rather than focusing on their limitations. This recommendation aligns with a systematic review by Strøm, Ytrehus and Grov (2016), which notes that limited attention has been given to evaluating communicative abilities in individuals with dementia, despite the crucial role communication plays in determining social connectedness, independence, and personal fulfilment. Implementing a standardized assessment tool for communication could assist nurses responding to individuals' needs, even amidst language barriers. To date, only two communication tools specifically for dementia have been developed: the Holden Communication Scale (Holden and Woods, 1995) and the Threadgold Communication Tool.

**Fig. 1:**
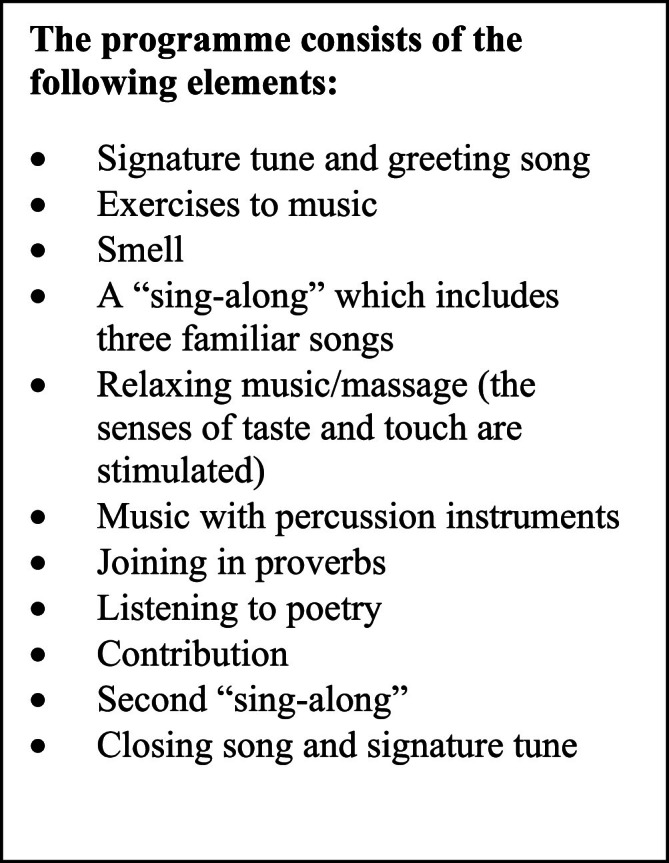
The Sonas Program.

The proxies that rate Threadgold Communication Tool are Sonas Licensed Practitioners leading or assisting at a Sonas session. Sonas Licensed Practitioners are trained in rating patients with the Threadgold Communication Tool. Following the psychometric evaluation of the English version of the Threadgold Communication Tool conducted on an Irish cohort (*n* = 51) of individuals with dementia (Strøm, Engedal, and Grov, 2016), modifications were implemented. Two elements, namely 'holding gaze' and 'following with gaze', were eliminated. However, the items 'smell' and 'taste' were incorporated to ensure a comprehensive evaluation of all 14 elements of the Sonas program and to include all senses (Fig. 1).

### 1.2 The Sonas program

The Sonas program is a multi-sensory stimulation program (Engaging Dementia, 2024). The program was developed by Sr. Mary Threadgold in 1990 (Sonas aPc, 2011). The program incorporates cognitive, sensory, and social stimulation, engaging all five senses: tactile, smell, taste, auditory, and visual. It serves as a therapeutic activity for individuals suffering from significant communication impairment, primarily due to dementia. The objectives of the Sonas program are threefold: (1) to activate whatever potential for communication has been retained by an older person with communication impairment, (2) to encourage the creation of an environment that will facilitate communication, and (3) to have activation of the potential for communication recognized and accepted as an essential part of care planning for older people. The programme consists of 13 elements and follows the same structure each time, believing that repetition is a way of helping the individual to remember (Sonas aPc, 2011). The participants are seated in a semi-circle and the session lasts 45 min, led by a person trained in the Sonas programme (Sonas Licensed Practitioners) and an assistant.

Until recent years, research on the Sonas program has been scarce despite its potential benefits, but today, four studies have been conducted. (Dolan et al., 2022; Goyal, Engedal, Benth, and Strøm, 2021; Strøm et al., 2017, 2018).

Approximately 300 nursing homes utilize the Sonas program in Ireland, with some adoption in England. There has also been an attempt to implement the program in Australia. Over the past three years, the Sonas program has been introduced at multiple locations in Norway, resulting in the certification of 130 healthcare professionals who now lead groups. Efforts are ongoing to further increase the program's recognition and adoption throughout Norway.

## 2 Method

### 2.1 Design

The study has a prospective design, where we investigated the psychometric properties of the Threadgold Communication Tool, Norwegian version, with two measurement points within 10 days.

### 2.2 Translation procedure

To translate and assess the ana of the instrument we used the first three steps in the protocol from the WHO: 1) translation, 2) expert panel, and 3) back-translation. The Threadgold Communication Tool was translated into Norwegian by two nurses and a psychologist from Norway, who were fluent in the English language. The three translations were collated by two people (a psychiatrist and a nurse) into the final Norwegian version, which was then translated back to English by a person fluent in both Norwegian and English language. This final version was compared with the original English version in a consensus meeting with the translators. The English back-translation version was sent to Sr. Threadgold, to get her comments. The final Norwegian version of the Threadgold Communication Tool was agreed upon after minor grammatical revisions and was thereafter used in this study.

### 2.3 Sample

The recommended sample size, as stated in methodological literature (Pallant, 2020), is 10 participants per item: in this case, with 13 items, resulting in a sample size of approximately 130 participants. We recruited 126 residents from ten different nursing homes and one assisted living facility in Norway for the implementation of the Sonas program. The participants consisted of 99 women (78.57 %) and 27 men (21.43 %), aged between 61 and 100 years, with a mean age of 85.67 (SD 7.59). The retest was administered to every second participant after two weeks, yielding responses from 69 residents. Inclusion criteria in the current study were being a participant in the Sonas program. Inclusion in the Sonas program requires diagnosis dementia or observed cognitive impairment, a criterion all participants met according to their medical records. Gender and age data were also obtained from the residents' records. The recruitment process took place between January and December 2023.

### 2.4 Data collection

Demographic data, age, and gender were collected at the same time as the completion of the Threadgold Communication Tool. To assess the test-retest reliability of the Threadgold Communication Tool, the instrument was administered twice within ten days, by the same Sonas Licensed Practitioners, as recommended by Polit and Beck (2020).

### 2.5 Statistical analysis

Data were analyzed using the SPSS, v. 27.0. Descriptive characteristics of the participants were performed, and there were no missing values. Internal consistency was examined by using Cronbach’s α coefficient and corrected item-total correlation. The criterion level for acceptable reliability was set to α ≥ 0.7 (Hardy and Bryman, 2004). To investigate if the items are measuring the same underlying characteristic, an inter-item correlation was performed. The test-retest reliability was examined with a paired sample *t*-test and intra-class correlation coefficient with a criterion level for acceptable set at ≥ 0.7. The suitability test of data for factor analysis was performed before the Explanatory Principal Component Analysis (PCA) based on Bartlett’s test of sphericity (significance >0.05) and the Kaiser Meyer Olkin (>0.60). Exploratory PCA was performed, including all 14 items of the Threadgold Communication Tool to explore the component structure and construct validity as this analysis is exploratory in its nature, aiming to uncover the latent structure of the data. The number of components retained for extraction was based on the Kaiser criterion (eigenvalue ≥ 1) as well as on an inspection of the scree plot. All samples were initially explored using Varimax rotation. Loadings >0.40 were considered satisfactory.

We have performed the study in accordance with the ‘COnsensus-based Standards for the Selection of Health Measurement Instruments’ (COSMIN) which includes recommendations for terminology, taxonomy, and methodology in studies concerning self-reporting questionnaires and their measurement properties (Mokkink et al., 2018).

### 2.6 Ethics

The study was approved by the responsible officials and heads of nursing homes. Informed consent for participation in the Sonas program and participation in research was obtained from the relatives of the participants in the Sonas group, as the persons with dementia were unable to sign the informed consent form themselves. There was no need to register with the National data protection agency, SIKT (849,595), nor for approval from the Regional Ethics Committee for Medical and Health Research in Southeast of Norway. This is because we did not collect personal or health data from individuals, and this study is a quality assurance study of the Threadgold Communication Tool, not a study examining the effect of the Sonas program or with new health outcomes as purpose.

## 3 Results

A total of 126 residents were included, with ages ranging from 61 to 100 years. The mean age for women was 85.57 (SD 7.81) and for men it was 86.04 (SD 6.81).

### 3.1 Internal consistency

Cronbach’s α coefficient for the total score was good, i.e. 0.95 (Table 1). The scores of the items in the Threadgold Communication Tool were normally distributed, with a mean of 2.55, and SD between 1.078 and 1.644. All the items had a Pearson’s correlation coefficient >0.15.

**Table 1:** Item performance for the Threadgold Communication Tool (*N* = 126).

Items		Mean	SD	Corrected item-total correlation	Cronbach’s α if item deleted
1	Speaking	2.66	1.375	.749	.941
2	Vocalization	2.03	1.644	.483	.950
3	Eye contact	3.21	1.078	.731	.943
4	Smiling	3.08	1.093	.753	.942
5	Singing	2.41	1.487	.752	.941
6	Using gesture	2.15	1.459	.760	.941
7	Interactive touch	2.45	1.446	.751	.941
8	Exercises	2.54	1.384	.865	.938
9	Rhythmic movements	2.39	1.420	.861	.938
10	Contribution	2.03	1.544	.782	.941
11	Using instruments	2.37	1.522	.770	.941
12	Appropriate posture	2.34	1.363	.779	.941
13	Smell	3.05	1.371	.577	.946
14	Taste	3.05	1.289	.625	.945

With such a high Cronbach’s α coefficient, a correlation analysis including the 14 items in the Threadgold Communication Tool and the total score was performed (Table 2). Only one correlation coefficient was >0.8: exercise and rhythmic movements (*r* = 0.830), which corresponds with the face validity of the TCT. The results show strong correlations between the Threadgold Communication Tool total score and the items: speaking (*r* = 0.779), eye contact (*r* = 0.743), smiling (*r* = 0.765), singing (*r* = 0.786), using gestures (*r* = 0.796), interactive posture (*r* = 0.784), exercises (*r* = 0.887), rhythmic movements (*r* = 0.883), contribution (*r* = 0.882), using instruments (*r* = 0.812) and appropriate touch (*r* = 0.811). A moderate correlation was found between the Threadgold Communication Tool total score and items' vocalization (*r* = 0.553), smell (*r* = 0.615), and taste (*r* = 0.657).

**Table 2:** Correlation matrix Threadgold Communication Tool at baseline (*N* = 126).

	Speaking	Vocalizing	Eye contact	Smiling	Singing	Using gesture	Interactive posture	Exercises	Rhythmic movements	Contributing	Using instrument	Appropriate touch	Smell	Taste	Total TCT
Speaking	1.000														
Vocalizing	.449	1.000													
Eye contact	.631	.321	1.000												
Smiling	.612	.327	.690	1.000											
Singing	.586	.371	.591	.582	1.000										
Using gesture	.575	.627	.542	.573	.578	1.000									
Interactive posture	.631	.358	.551	.613	.507	.622	1.000								
Exercises	.667	.430	.682	.662	.701	.680	.674	1.000							
Rhythmic movements	.650	.408	.697	.696	.692	.697	.709	.830	1.000						
Contributing	.565	.479	.528	.628	.655	.668	.605	.748	.709	1.000					
Using instruments	.577	.229	.623	.621	.738	.583	.559	.793	.776	.710	1.000				
Appropriate touch	.569	.396	.602	.609	.620	.625	.725	.660	.736	.633	.647	1.000			
Smell	.498	.249	.414	.434	.445	.355	.470	.504	.471	.412	.419	.447	1.000		
Taste	.507	.243	.503	.523	.450	.420	.509	.525	.491	.431	.467	.522	.783	1.000	
Total TCT	.779	.553	.743	.765	.786	.796	.784	.887	.883	.882	.812	.811	.615	.657	1.000

The inter-item correlation matrices were all positive; three items, ‘vocalization’, ‘smell’, and ‘taste’ scored below 0.7. The corrected item-total correlation ranged between 0.5 and 0.9, with a mean score of 0.7 (Table 2).

### 3.2 Test-retest reliability

The two-time measure points' results did not differ significantly (paired sample *t*-test, *t* = 0.5, *p* = 0.6, two-sided). The intra-class correlation between the two assessments was strongly and positively correlated (*r* = 0.8, *p* < 0.001). The Threadgold Communication Tool total mean score was 35.30 (SD 15.74) at baseline and 34.72 (SD 15.56) at retest (Table 3).

**Table 3:** Test-retest reliability measures for the Threadgold Communication Tool (*n* = 69).

Scale		Baseline test (*n* = 69) mean (SD)	Retest (*N* = 69) mean (SD)	p value
1	Speaking	2.68 (1.42)	2.46 (1.52)	< 0.218
2	Vocalization	2.23 (1.59)	2.00 (1.72)	< 0.145
3	Eye contact	3.17 (1.08)	3.17 (1.01)	1.000
4	Smiling	3.00 (1.20)	2.91 (1.29)	< 0.450
5	Singing	2.46 (1.42)	2.39 (1.46)	< 0.573
6	Using gesture	2.28 (1.41)	2.23 (1.45)	< 0.738
7	Interactive touch	2.33 (1.44)	2.30 (1.41)	< 0.791
8	Exercises	2.43 (1.34)	2.48 (1.34)	< 0.732
9	Rhythmic movements	2.35 (1.47)	2.48 (1.28)	< 0.327
10	Contribution	1.90 (1.56)	2.04 (1.62)	< 0.248
11	Using instruments	2.28 (1.49)	2.41 (1.35)	< 0.309
12	Appropriate posture	2.38 (1.38)	2.68 (1.30)	< 0.034
13	Smell	2.61 (1.53)	2.55 (1.61)	< 0.650
14	Taste	2.81 (1.48)	2.77 (1.51)	< 0.772
Total score		**35.30 (15.74)**	**34.72 (15.56)**	**< 0.001**

Statistics: Paired samples test.

### 3.3 Principal component analysis

The Explanatory Principal Component Analysis (PCA) revealed a two-component structure when the eigenvalue ≥ 1 was used and the scree plot was inspected. We first examined the suitability for conducting a PCA of the Threadgold Communication Tool by inspecting the correlation matrix (Table 2). Bartlett’s test of sphericity reached statistical significance (*p* =<0.001), and the Kaiser Meyer Olkin was 0.90. Based on this, a PCA was found to be appropriate.

The first component consisted of the following items: speaking, vocalization, eye contact, smiling, singing, using gestures, interactive posture, exercises, rhythmic movements, contribution, using instruments, appropriate touch, and taste. It accounted for 62.9 % of the variance. The second factor consisted of one item; smell and accounted for 7.6 % of the variance. When performing a rotated component matrix using Varimax with Kaiser Normalization, the second component consisted of two items; smell and taste (Table 4), and accounted for 24.5 % of the variance, while factor one accounted for 46 % of the variance.

**Table 4:** Explanatory PCA with rotation of the 14 items of the Threadgold Communication Tool (*n* = 126).

	Component matrix		Rotated component matrix	
	Component 1	Component 2	Component 1	Component 2
Speaking	**.786**	.033	.**637**	.462
Vocalization	**.528**	−0.441	**.684**	−0.076
Eye contact	**.776**	.050	**.619**	.471
Smiling	**.795**	.042	**.639**	.475
Singing	**.793**	−0.066	**.697**	.383
Using gesture	**.790**	−0.325	**.838**	.166
Interactive touch	**.792**	.023	**.648**	.457
Exercises	**.892**	−0.063	**.778**	.440
Rhythmic movements	**.894**	−0.092	**.796**	.418
Contributing	**.817**	−0.208	**.796**	.278
Using instruments	**.821**	−0.029	**.700**	.430
Interactive posture	**.817**	−0.026	**.696**	.430
Smell	.622	**.634**	.186	**.872**
Taste	.**668**	.611	.219	**.879**
Total TCT	.992	−0.023	.839	.529
Explained variance	**62.9%**	**7.6%**	**46%**	**24.5%**

Bold formatting is used to indicate the items belonging with components 1 or 2.

## 4 Discussion

The aim of this study was to investigate the psychometric properties of the translated version of the Threadgold Communication Tool in a Norwegian sample using the Norwegian version of the Threadgold Communication Tool. The sample consisted of 126 persons, with a mean age of 85.67 (SD 7.59) years, mainly women (78.57 %), which complies with the demographic in Norwegian nursing homes (Gjøra, Kjelvik, Strand, Kvello-Alme og Selbæk, 2020). To our knowledge, this is the first study to investigate the psychometric properties of the Norwegian version of the Threadgold Communication Tool.

Cronbach’s α coefficient for the total Threadgold Communication Tool was 0.95. The same high coefficient was found by Strøm et al. (2016) for the total score when conducting the initial psychometric evaluation of the English version of the Threadgold Communication Tool. This indicates a satisfactory level of internal consistency, indicating that most items measure the same construct. This is further supported by the Pearson’s correlation coefficients, which were all above 0.5, and the fact that the inter-item correlation matrices were all positive, and the results of the PCA. Except for two items all other items clustered in the same component. These findings indicate that the 14 items in the Threadgold Communication Tool measure the same underlying characteristics (Pallant, 2020). Only the correlation coefficient between exercise and rhythmic movements (*r* = 0.8) was considered high (*r*= >0.8) which is as expected since these two concepts are considered to be somewhat the same and match the feedback given by the Sonas Licensed Practitioners. Three items, ‘vocalization’, ‘smell’, and ‘taste’ scored below 0.7; however, all were still above 0.3, indicating a moderate correlation.

The result of the test-retest analyses yielded satisfactory values for the total score of Threadgold Communication Tool. A stable correlation was observed between the baseline and the two-week mark, with no significant difference. This stability was also evident in the comparison between individual items, indicating a robust test-retest reliability. This shows that the assessments were carried out in a consistent way by the Sonas Licenced Practitioner.

The results from the correlation matrix show strong correlations between the Threadgold Communication Tool total score and the items “speaking”, “eye contact”, “smiling”, “singing”, “using gestures”, “interactive posture”, “exercises”, “rhythmic movements”, “contribution”, “using instruments”, and “appropriate touch”. A corrected item-total correlation analysis showed a score between 0.5 and 0.9. The lowest score was ‘vocalization’, and as in the validation of the English version of Threadgold Communication Tool from 2016 (Strøm et al., 2016), this item scored lower than the rest of the items. In the current psychometric testing, ‘vocalization’ is the only item we can remove without Cronbach’s α coefficient dropping. This also corresponds with face validity of the TCT. The Sonas Licensed Practitioners may have different understandings of the item, and it is important that the understanding of ‘vocalizing’ in the Threadgold Communication Tool is clarified before considering removing it from the scale. The raters should have consensus on what is meant and what is understood by the term ‘vocalization’. The Sonas Licensed Practitioners state that this item is difficult and does not make sense when scoring the instrument. When residents lack words, what they wish to express themselves, must be conveyed in a different manner. The challenge is that if one resident scores on ‘speaking’, the same resident do not score on ‘vocalization’ – and it becomes an either-or situation, which affect the total score and may result in an "incorrect" assessment of the degree of communication capacity. An outline of what ‘vocalization’ means in this instrument is needed in addition to the description in the manual.

In this study, two items, 'smell' and 'taste', were included in the Threadgold Communication Tool to ensure that it encompasses all 14 elements of the SONAS programme. These items replaced two others that were removed after the psychometric testing of the English version of the Threadgold Communication Tool (Strøm et al., 2016). This is the first psychometric analysis to include these two items.

The remaining items, including 'speaking', 'vocalization', 'eye contact', 'smiling', 'singing', 'using gestures', 'interactive posture', 'exercises', 'rhythmic movements', 'contribution', 'using instruments', and 'appropriate touch', formed a single component. This component represents both verbal and non-verbal communication, a structure that differs from the English version of the Threadgold Communication Tool (Strøm et al., 2016), which separates verbal and non-verbal communication.

This difference may be attributed to the increasing difficulty in interpreting facial expressions over time, presenting a challenge for Sonas Licensed Practitioners to detect these subtle cues. Additionally, it has been observed that patients often 'awaken' when they smell or taste something familiar. This raises questions about the presentation of these sensory stimuli and whether sufficient time is allowed for a response to occur. It also suggests that Sonas Licensed Practitioners using the Threadgold Communication Tool may need to focus more on the patient's response to taste and smell, rather than simply whether the patients can taste or smell.

Threadgold Communication Tool is made to assess communication capacity in patients in the SONAS program. Further, we recommend examining the possibility that, with adjustments to the Threadgold Communication Tool, the form could be utilized to assess general communication capacity in patients with dementia outside of the SONAS program. Currently, as far as we know no standardized form exists for evaluating the communication capacities of individuals with advanced dementia, making this a potential important adaptation.

## 5 Conclusion

The Norwegian version of the Threadgold Communication Tool demonstrates satisfactory psychometric properties, with a high level of internal consistency and robust test-retest reliability. However, the item 'vocalization' presents some challenges, scoring lower than other items and being identified as difficult to interpret by the Sonas Licensed Practitioners. This suggests a need for further clarification of this item.

The inclusion of 'smell' and 'taste' in the Threadgold Communication Tool is a novel aspect of this study, and the results indicate that these items fit well within the instrument's structure. However, the high correlation between some items suggests some redundancy. While some degree of redundancy can help to ensure the reliability of the scale, excessive redundancy can make the scale unnecessarily long and repetitive, potentially leading to disengagement. Hence, a version devoid of overlapping items is suggested for future development, and this issue may require attention in subsequent iterations of the Threadgold Communication Tool. Overall, the study contributes valuable insights to the field, particularly given the lack of instruments in Norwegian language. However, further research is needed to validate the Threadgold Communication Tool towards other communication tools and to continue refining the instrument based on these findings.

The implication of this study establishes the Norwegian version of the Threadgold Communication Tool as a reliable instrument with strong psychometric properties for assessing communication in dementia care. The findings serve as a foundation for further research and refinement, enhancing communication assessment resources available in the Norwegian language.

## CRediT authorship contribution statement

**Anne-Martha Utne Øygarden:** Writing – original draft, Visualization, Formal analysis, Conceptualization. **Ellen Karine Grov:** Writing – review & editing, Validation, Supervision, Methodology, Formal analysis, Conceptualization. **Anne Marie Mork Rokstad:** Writing – review & editing, Supervision. **Orla Brady:** Writing – review & editing, Supervision. **Knut Engedal:** Writing – review & editing, Supervision. **Benedicte Sørensen Strøm:** Writing – review & editing, Visualization, Project administration, Formal analysis, Conceptualization.

## Declaration of competing interest

The authors declare that they have no conflicts of interest.

## Appendix Supplementary materials

Image, application 1
