# Supplementary material for: Psychometric evaluation of the Norwegian version of the Threadgold Communication Tool
Source: Int J Nurs Stud Adv. 2025 Aug 6;9:100405. doi: 10.1016/j.ijnsa.2025.100405 (PMC12410571; doi:10.1016/j.ijnsa.2025.100405)
Supplement: Supplementary file 1 [file mmc1.pdf]

Vedlegg 5

Threadgold kommuniksjonsvurderingsverktøy (TCT)

Threadgold Kommunikasjonsverktøy måler kommunikasjonsevne under en Sonas gruppesamling. Den er primært utviklet for eldre personer med demens (Engaging Dementia, 2018). Sonas gruppesamling gjennomføres av sertifiserte Sonas gruppeleder). TCT består av 14 deler, hvor hver del skåres på en skala fra 0-4. Poengene måler observert kommunikasjonsevner under Sonas gruppesamling. Jo høyere skåre, jo høyere kommunikasjonsevner. Høyeste sammenlagte poengskåre er 56.

Ingen tegn på kommunikasjonsevne = 0, veldig små tegn på kommunikasjonsevne = 1, noen tegn på kommunikasjonsevne= 2, regelmessige tegn på kommunikasjonsevne = 3, hyppige tegn på kommunikasjonsevne = 4.

Poengsummer under 14 = alvorlige kommunikasjonsutfordringer, 15-25 = moderate kommunikasjonsutfordringer, 26-44 = milde kommunikasjonsutfordringer, 45-56 = ingen kommunikasjonsutfordringer.

Navn på deltaker: ______________________________________

1. Snakker	0	1	2	3	4
2. Lager verbale lyder	0	1	2	3	4
3. Har blikkontakt	0	1	2	3	4
4. Smiler	0	1	2	3	4
5. Synger	0	1	2	3	4
6. Bruker gester	0	1	2	3	4
7. Viser interaktiv berøring	0	1	2	3	4
8. Deltar i øvelser	0	1	2	3	4
9. Viser rytmiske bevegelser	0	1	2	3	4
10. Bidrar	0	1	2	3	4
11. Bruker instrumenter	0	1	2	3	4
12. Viser en interaktiv holdning	0	1	2	3	4
13. Responderer på lukt	0	1	2	3	4
14. Responderer på smak	0	1	2	3	4

Delsum:

Sammenlagt skår (av 56): _____

Dato: _________
Navn på Sonas gruppeleder): ______________________

Antallet deler er ble økt fra 12 til 14 i denne utgaven av TCT (2018) for å inkludere sansene lukt og smak, basert på resultatene av den psykometriske analysen utført i 2016 (Strøm, Engedal & Grov, 2016).

Verbal kommunikasjon
1. *Snakker:* uttrykker ideer eller følelser i ord, eksempler på verbal interaksjon sees
2. *Lager verbale lyder:* uttrykker seg kun med lyd, ingen identifiserbare ord, men hvilken som helst lyd uttalt av deltakeren, for eksempel latter.

Ikke-verbal kommunikasjon
3. *Har blikkontakt:* møter kort øynene til en annen person, enten gruppelederen, hjelperen eller et annet medlem av gruppen.
4. *Smiler:* bruker lepper/munn for å formidle glede eller tilfredshet.
5. *Synger:* viser evne til å synge ordene, for eksempel 'la, la' eller nynning under denne delen av samlingen.
6. *Bruker gester:* enhver gest som kan tolkes som å være utført bevisst, for eksempel at personen vinker, peker, dirigerer musikken eller gir tegn til å ikke ville ha noe.
7. *Viser interaktiv berøring:* for eksempel at personen klapper eller stryker andres hånd, eller holder hender. Kan observeres når du håndhilser under hilsen og farvel.
8. *Deltar i øvelser:* deltar i hele eller deler av de fysiske øvelsene etter beste evne.
9. *Viser rytmiske bevegelser:* bruke rytmeinstrument i takt med musikken, bevege seg etter musikk, som å klappe eller trampe takten, tappe takten med fingrene, alt etter personenes evne.
10. *Bidrar:* tegn på vilje til å prøve å synge, si et dikt eller annet personlig bidrag, enten verbalt eller non-verbalt eller at personen blir oppfordret av Sonas gruppeleder til å bidra f.eks. ved at Sonas gruppeleder forteller personens historie.
11. *Bruker instrumenter:* enhver aktiv bruk av et instrument
12. *Viser en interaktiv holdning:* åpen holdning, vender seg mot noen, tar noens hånd. F.eks. ser personen ut til å være engasjert under samlingen, hvordan reflekterer deres kroppsspråk deres engasjement.
13. *Responderer på lukt:* respons på stimulering av lukt.
14. *Responderer på smak*: respons på stimulering av smak.

Referanser

Sonas apc. (2018). Sonas Course Booklet.

Strøm, B. S., Engedal, K., & Grov, E.-K. (2016). A Psychometric Evaluation of the Threadgold Communication Tool for Persons with Dementia. Dementia and Geriatric Cognitive Disorders Extra, 6(1), 150-160.

Sonas apc 2018
